# Generation of pure lymphatic endothelial cells from human pluripotent stem cells and their therapeutic effects on wound repair

**DOI:** 10.1038/srep11019

**Published:** 2015-06-12

**Authors:** Shin-Jeong Lee, Changwon Park, Ji Yoon Lee, Sangsung Kim, Pil Jae Kwon, Woansang Kim, Yong Heui Jeon, Eugine Lee, Young-sup Yoon

**Affiliations:** 1 Division of Cardiology, Department of Medicine; 2 Department of Pediatrics, Emory University School of Medicine, Atlanta, GA, 30322, USA; 3 Severance Biomedical Science Institute, Yonsei University College of Medicine, Seoul, Korea

## Abstract

Human pluripotent stem cells (hPSCs) have emerged as an important source for cell therapy. However, to date, no studies demonstrated generation of purified hPSC-derived lymphatic endothelial cells (LECs) and tested their therapeutic potential in disease models. Here we sought to differentiate hPSCs into the LEC lineage, purify them with LEC markers, and evaluate their therapeutic effects. We found that an OP9-assisted culture system reinforced by addition of VEGF-A, VEGF-C, and EGF most efficiently generated LECs, which were then isolated via FACS-sorting with LYVE-1 and PODOPLANIN. These hPSC-derived LYVE-1^+^PODOPLANIN^+^cells showed a pure committed LEC phenotype, formed new lymphatic vessels, and expressed lymphangiogenic factors at high levels. These hPSC-derived LECs enhanced wound healing through lymphangiogenesis and lymphvasculogenesis. Here we report, for the first time, that LECs can be selectively isolated from differentiating hPSCs, and that these cells are potent for lymphatic vessel formation *in vivo* and wound healing. This system and the purified hPSC-derived LECs can serve as a new platform for studying LEC development as well as for cell therapy.

Lymphatic vessels play an important role in tissue fluid homeostasis and immune surveillance, and thus dysfunctions in lymphatic vessels lead to the development of diseases such as lymphedema and tumors. Despite a continuous increase in lymphatic disorders, current therapeutic options for modifying lymphatic pathophysiology are very limited. Recent progress in the field of lymphatic development has enhanced our understanding of molecular regulation of lymphatic vessel formation. In developing mouse embryos, LECs differentiate from a subpopulation of the endothelial cells of the cardinal vein and subsequently form the mature lymphatic vasculature with coordinated expression of SOX-18, PROX-1, LYVE-1, VEGFR3/VEGFC and PODOPLANIN^1^,^2^,^3^,^4^,^5^,^6^. More recently, attempts have been made to develop *in vitro* lymphatic differentiation systems using embryonic stem cells (ESCs) to establish a model system to investigate lymphatic vascular differentiation and to obtain a targeted cell population for therapeutic application. In addition, the discovery of induced pluripotent stem cells (iPSCs) has increased interest in using hPSCs, i.e., human embryonic stem cells (hESCs) and human induced pluripotent stem cells (hiPSCs), for cell therapy^7^,^8^,^9^,^10^,^11^.

Studies have reported the feasibility of lymphatic endothelial lineage differentiation from mouse pluripotent stem cells (mPSCs). Liersch *et al*.^12^ demonstrated that initial emergence of cells with a LEC phenotype can be detected in embryoid bodies (EBs) at day 18 of spontaneous differentiation of mESCs. Kreuger *et al*.^13^ also showed that mESCs which were differentiated through EBs for 4 days and further cultured on type I collagen-coated plates expressed lymphatic markers. Although these studies have shown lymphatic endothelial differentiation characteristics of mESCs or miPSCs, they did not show detailed expression kinetics of LEC development or to confirm the identity of the PSC-derived cells as LECs *in vivo*. To date, no studies were reported regarding the *in vivo* behavior and therapeutic potential of hPSC-derived LECs.

A very common, but not widely known as lymphatic-related, disorder is skin wound. Wound healing is a complex process including coagulation, inflammation involving recruitment of inflammatory cells into the injured sites, and formation of granulation tissue with angiogenesis and lymphangiogenesis, followed by a remodeling process^14^. Impaired wound healing often becomes a serious complication in several diseases including diabetes. Recent studies have shown the importance of lymphatic vessel regeneration in wound healing. In cutaneous wound healing models of pig and mouse, VEGFR3-expressing lymphatic vessels were found in the granulation tissue followed by regression at later stages^15^,^16^. VEGFC, a ligand for VEGFR3, was increased in response to tissue injury^17^. While augmented expression of VEGFC can significantly promote wound healing as well as lymphangiogenesis, inhibiting VEGFC or another VEGFR3 ligand, VEGFD, leads to delayed recovery of wound^17^. Furthermore, macrophages derived from diabetic mice failed to improve wound repair, but upon activation with IL-1β promoted the recovery of the tissue injury with enhanced lymphatic regeneration, suggesting a critical role of lymphatic vessels in wound healing^18^. Despite this emerging knowledge of the importance of lymphatic vessels in wound healing, there are no studies available regarding the effects of stem cell therapy targeting lymphatic neovascularization on wound repair.

In this study, we for the first time developed an efficient culture system to differentiate hESCs and hiPSCs into the lymphatic endothelial lineage and isolated LYVE-1^+^PODOPLANIN^+^cells as functional LECs. Furthermore, we demonstrated the contribution of these hPSC-derived LECs to *in vivo* lymphatic vascular commitment and their therapeutic potential in wound healing.

## Results

### Generation of cells expressing lymphatic markers from hESCs and hiPSCs

**Figure 1:**
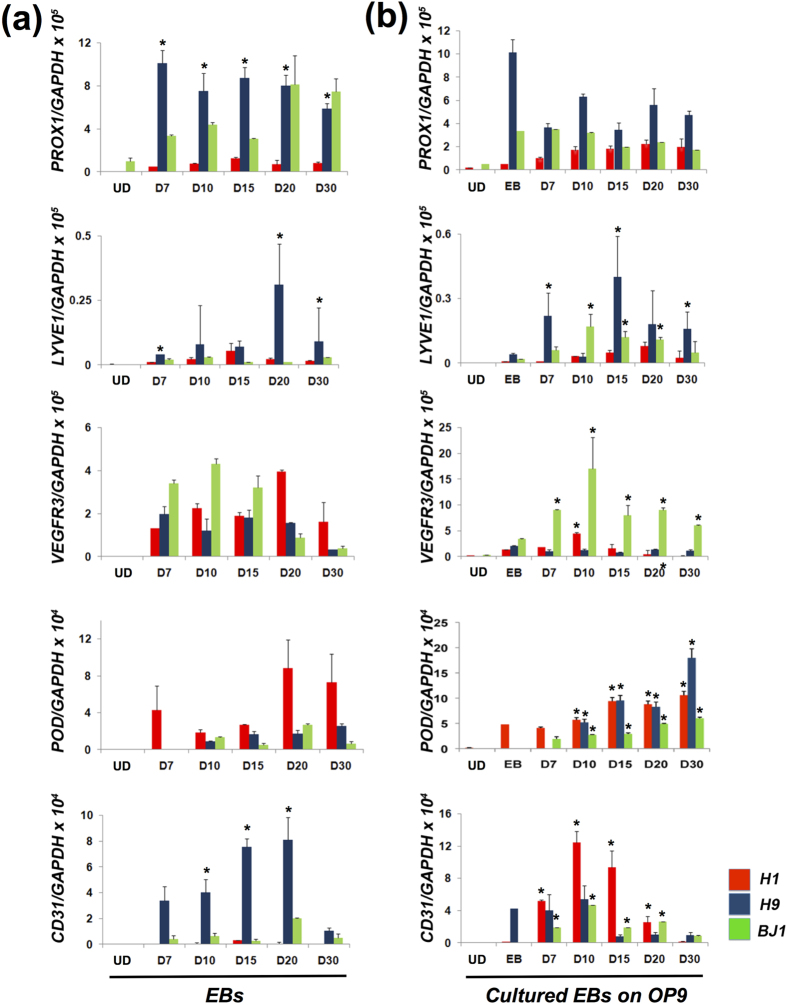
Differentiation of hESCs (H1 and H9) and hiPSCs (BJ1) into the LEC lineage. (**a**) qRT-PCR analyses of *in vitro* differentiated H1, H9 and BJ1 cells through EB formation. N = 9 per group. *P < 0.05 vs. H1. (**b**) qRT-PCR analyses of *in vitro* differentiated H1, H9 and BJ1 cells on OP9 cells with lymphangiogenic cytokines. EBs differentiated for 7 days in suspension culture were replated on OP9 cells, further cultured for an additional 30 days with VEGF-A, -C and EGF, and subjected to qRT-PCR. N = 9 per group. *P < 0.05 vs. EB. UD: Undifferentiated hPSCs, POD: PODOPLANIN.

Since no studies have demonstrated generation of pure LECs from human pluripotent stem cells (hPSCs), we first sought to establish an efficient LEC differentiation system by trying three different culture conditions: spontaneous differentiation through EB formation, co-culture with OP9 cells, and a feeder-free culture with gelatin for lymphatic endothelial differentiation of hESC lines (H1 and H9) and hiPSCs (BJ1)^19^. First, the pluripotent cells were induced to form EBs and cultured under suspension conditions for 30 days. To determine whether hESCs were differentiated into LECs, we performed gene expression analysis with an emphasis on the expression of key LEC markers such as *PROX1, LYVE1, VEGFR3 and PODOPLANIN*. We found that hESCs (H1 and H9) began to express the LEC markers at day 7, peaked around day 20, and maintained expression up to day 30 (Fig. 1a). SOX18 and COUP-TFII, markers for early lymphatic endothelial cell specification, were also expressed in EB to D30 (see Supplementary Fig. S1 online). A similar expression pattern was observed in hiPSCs (BJ1, Fig. 1a) and in another hiPSC line that we generated from human dermal fibroblasts (hiPS-E1 line, data not shown). Interestingly, gene expression analysis revealed that expression of *PROX1*, *LYVE1*, and *CD31* were significantly lower in H1 than in H9, suggesting variable differentiation potential between hESC lines^20^. In addition, we observed that the kinetics of LEC gene expression differed slightly between hESCs and hiPSCs, indicating intrinsic variation in differentiation characteristics of these cells^21^.

Next, we used a combination of lymphangiogenic cytokines and OP9 cells as a feeder layer for lymphatic endothelial differentiation^22^,^23^. EBs differentiated for 7 days under suspension conditions were subsequently cultured on OP9 cells for an additional 30 days with VEGF-A, VEGF-C and EGF^13^,^24^,^25^. EGF was added to prevent cell death and augment cell proliferation. Overall, this culture condition induced expression of the LEC markers in all hESC and hiPSC lines at higher levels than spontaneous differentiation (Figs 1a,b). Compared to the spontaneous differentiation system, *LYVE1* and *VEGFR3* expression were more highly detected in BJ1, and *PODOPLANIN* expression was 3- to 15-fold higher in H9 and BJ1 under these co-culture conditions. OP9 cells did not express LEC markers (see Supplementary Fig. S2 online). Together, these data indicate that a culture system using OP9 cells, lymphangiogenic cytokines, and EGF can induce lymphatic endothelial differentiation from hESCs and hiPSCs to a greater extent than spontaneous differentiation, and furthermore suggest that this system can reduce variation of lymphatic endothelial differentiation capacity between human pluripotent cell lines.

Finally, we tried a feeder-free system for LEC differentiation in which the cells were cultured onto gelatin with the same lymphatic growth factors and EGF. However, this condition resulted in lower efficiency for LEC differentiation and higher cell death compared to the OP9 cell assisted culture system (data not shown). Collectively, our results clearly indicated the successful differentiation of hPSCs into the LEC lineage.

### Characterization of hPSC-derived LECs

**Figure 2:**
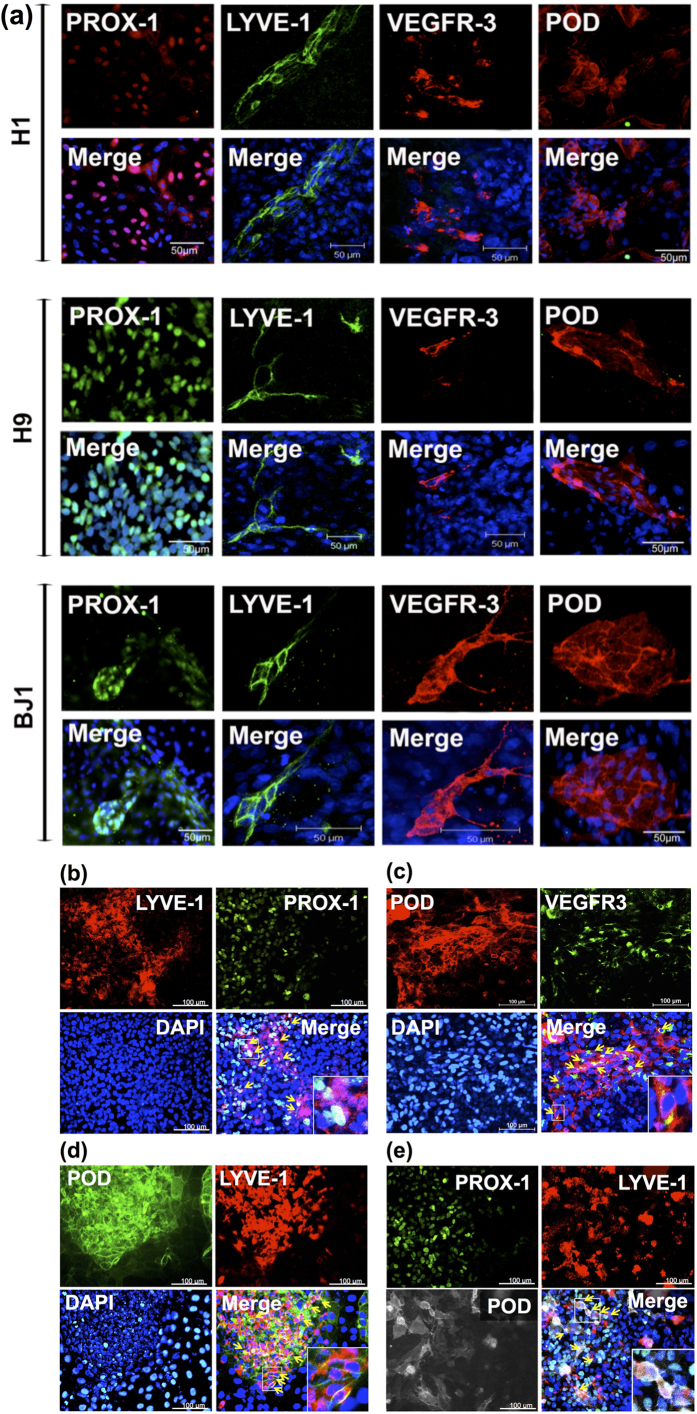
Immunocytochemistry showing expression of LEC markers in the differentiated hESCs and hiPSCs. The pluripotent stem cells differentiated in suspension culture were subsequently cultured for 10 to 15 days under the co-culture conditions. (**a**) Single LEC marker staining for LYVE-1, VEGFR3, or PODOPLANIN (POD). DAPI: blue. (**b-d**) Double LEC marker staining LYVE-1 and PROX-1 (**b**), VEGFR-3 and PODOPLANIN (**c**), and PODOPLANIN and LYVE-1 (**d**) in H9-derived LECs. (**e**) Triple LEC marker staining LYVE-1, PODOPLANIIN, PROX-1 in H9-derived LECs. Arrows indicate the representative cells stained positive for two or three LEC markers as labeled.

To further characterize the identity of the cells derived from hESCs (H1 and H9) and hiPSCs (BJ1), we investigated whether the differentiated cells expressed LEC markers at the protein level with proper localization. In LECs, PROX-1, a transcription factor, is expressed in the nucleus, whereas LYVE-1, VEGFR3 and PODOPLANIN are localized in the cell membrane. As expected, immunostaining results showed (Fig. 2a) that while PROX-1 was exclusively localized in the nuclei of the differentiated cells, LYVE-1, VEGFR3 and PODOPLANIN were found in cell membranes, confirming correct localization of the key LEC markers in the differentiated cells. We also confirmed co-expression of double or triple LEC markers in differentiating cells (Figs 2b–e, Supplementary Fig. S3 and S4 online).

**Figure 3:**
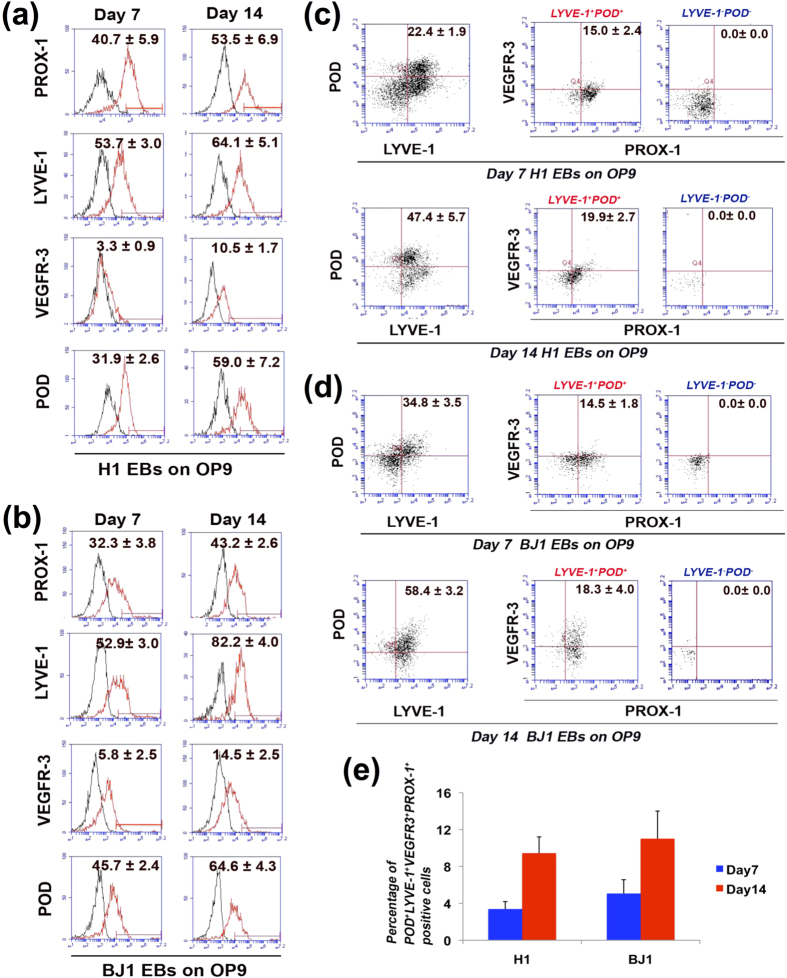
FACS analyses for multiple LEC marker expression in differentiated hESCs and hiPSCs. FACS analyses were performed with H1 and BJ1 cells differentiated under the co-culture conditions for 7 and 14 days. (**a**, **b**) Single LEC marker staining for H1 (**a**) and BJ1 (**b**). (**c**, **d**) Quadruple staining with LEC markers for H1 (**c**) and BJ1 (**d**). LYVE-1^+^POD^+^and LYVE-1^−^POD^−^cells were re-plotted for both VEGFR-3 and PROX-1. (**e**) Bar graph for average percentage of differentiated H1 and BJ1 cells positive for four LEC markers. Number in each box of A through D represents percentage of positive cells of each indicated protein. N = 3, POD: PODOPLANIN.

Next, we further examined the lymphatic endothelial differentiation of hESCs and hiPSCs with FACS analysis (Fig. 3). FACS analysis of H1 and BJ1-derived cells showed that after 14 days of co-culture with OP9 cells and lymphangiogenic growth factors, about 40–70% of these cells expressed PROX-1, LYVE-1 or PODOPLANIN, and about 10–15% expressed VEGFR3 (Figs 3a,b). Cells double-positive for LYVE-1 and PODOPLANIN, VEGFR3 and PODOPLANIN, or LYVE-1 and VEGFR3 were observed at 10–50% at day 14 (data not shown). Next, we used the four LEC markers for FACS to further verify the identity of the cells, and found that about 10% of the differentiated cells at day 14 expressed all four LEC markers (Figs 3c–e), suggesting the LEC identity of differentiated H1 and BJ1 cells in our culture system. Since macrophages can also express LYVE-1 or PODOPLANIN^26^,^27^, we also performed flow cytometry with CD14, a marker for human monocyte-macrophage lineage cells and found that virtually no LYVE-1^+^PODOPLANIN^+^cells were positive for CD14, indicating that these cells were not monocyte-macrophage lineage cells (see Supplementary Fig. S5 online). It is of note that under these culture conditions, the lymphatic endothelial phenotype was effectively maintained and the number of these cells increased for more than two weeks. We conclude that this culture system can effectively differentiate hESCs and hiPSCs into the LEC lineage.

### LYVE-1^+^PODOPLANIN^+^cells differentiated from hPSCs contribute to lymphatic vessel formation

**Figure 4:**
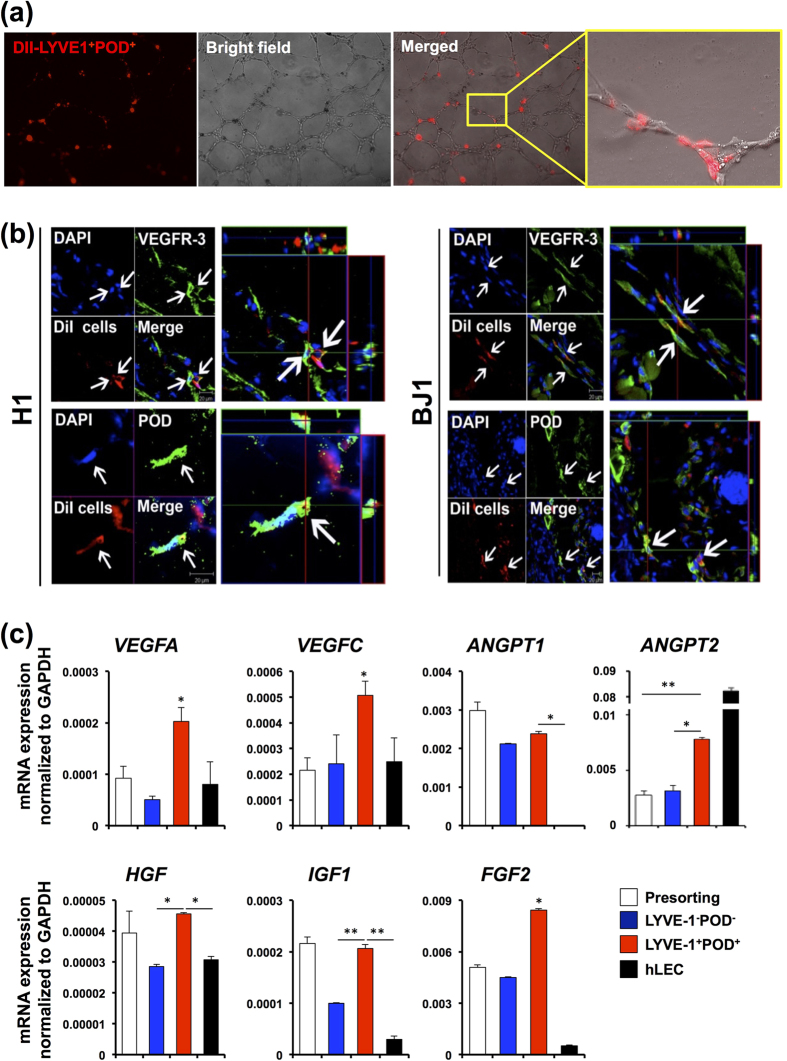
Contribution of LYVE-1^+^PODOPLANIN^+^cells derived from hESCs and hiPSCs to new lymphatic vessel formation. (**a**) *In vitro* tube formation assay. DiI-labeled LYVE-1^+^PODOPLANIN^+^cells (red) derived from hiESC (H1) cocultured with hLECs onto Matrigel for 12 hours, showing their incorporation into vascular structures consisting of hLECs. (**b**) DiI-labeled LYVE-1^+^PODOPLANIN^+^cells which were differentiated from H1 and BJ1 cells were injected into mice in an ear wound model. Two weeks later, tissues were harvested and subjected to immunohistochemistry with VEGFR3, PODOPLANIN, and LYVE-1 antibodies on frozen tissue sections. Confocal microscopic imaging revealed incorporation of injected cells (red) into lymphatic vascular structures and expression of PODOPLANIN and VEGFR3. (**c**) mRNA expression of the indicated lymphangiogenic genes in pre-sorted cells at day 14, LYVE1^+^PODOPLANIN^+^cells (BJ1-derived), LYVE1^−^PODOPLANIN^−^cells (BJ1-derived), and hLECs measured by qRT-PCR. Data were presented as relative mRNA expression to GAPDH. N = 3 to 4 per group. ^*^P < 0.05. ^**^P < 0.01.

To validate the identity and functionality of the lymphatic endothelially committed cells isolated from hESCs and hiPSCs differentiated under our culture conditions, we performed a series of *in vitro* and *in vivo* studies. *In vivo* behavior of lymphatic-endothelially differentiated ESCs or iPSCs has not been previously reported for human or mouse cells^12^,^13^,^25^,^28^. As PROX-1 is a nuclear protein, which is not suitable for cell sorting experiments without affecting cell viability, we took advantage of the two surface markers of LECs, LYVE-1 and PODOPLANIN to enrich LEC lineages from differentiated hPSCs. First, we tested *in vitro* activities of hESC- or hiPSC-derived LECs using a tube formation assay. Cells positive for both LYVE-1 and PODOPLANIN were isolated by FACS from differentiating H1 and BJ1 cells at day 14, labeled with a red fluorescent dye, CM-DiI^29^, and subjected to tube formation assay. As shown in Supplementary Fig. S6, the sorted cells readily formed tube-like structures. Further, the LYVE-1^+^PODOPLANIN^+^cells were able to incorporate into the tubular structure upon co-culture with hLECs in a Matrigel tube formation assay (Fig. 4a). Next, to determine the vasculogenic potential of hPSC-derived LECs *in vivo*, we injected the sorted cells into an ear wound model in which lymphatic vessel growth occurs^18^,^30^, harvested the tissues at 2 weeks, and conducted immunohistochemistry with frozen sections. A confocal microscopic examination of the stained sections with three-dimensional reconstruction demonstrated that the injected LYVE-1^+^PODOPLANIN^+^cells were incorporated into lymphatic vessels and expressed LEC markers (Fig. 4b). Finally, we determined lymphangiogenic gene expression in LYVE-1^+^PODOPLANIN^+^cells. We measured mRNA expression of *VEGFA, VEGFC, ANGPT1, ANGPT2, HGF, IGF*, and *FGF2* by qRT-PCR. Surprisingly, all the measured genes except *ANGPT2* were more highly expressed in LYVE1^+^PODOPLANIN^+^cells compared to LYVE1^-^PODOPLANIN^−^cells and hLECs. These experiments clearly suggested that the LYVE-1^+^PODOPLANIN^+^cells have pro-lymphangiogenic properties and have the capability for lymphatic vessel formation as LECs *in vivo*.

### LYVE-1^+^PODOPLANIN^+^cells differentiated from hPSCs promote wound healing through lymphatic neovascularization

**Figure 5:**
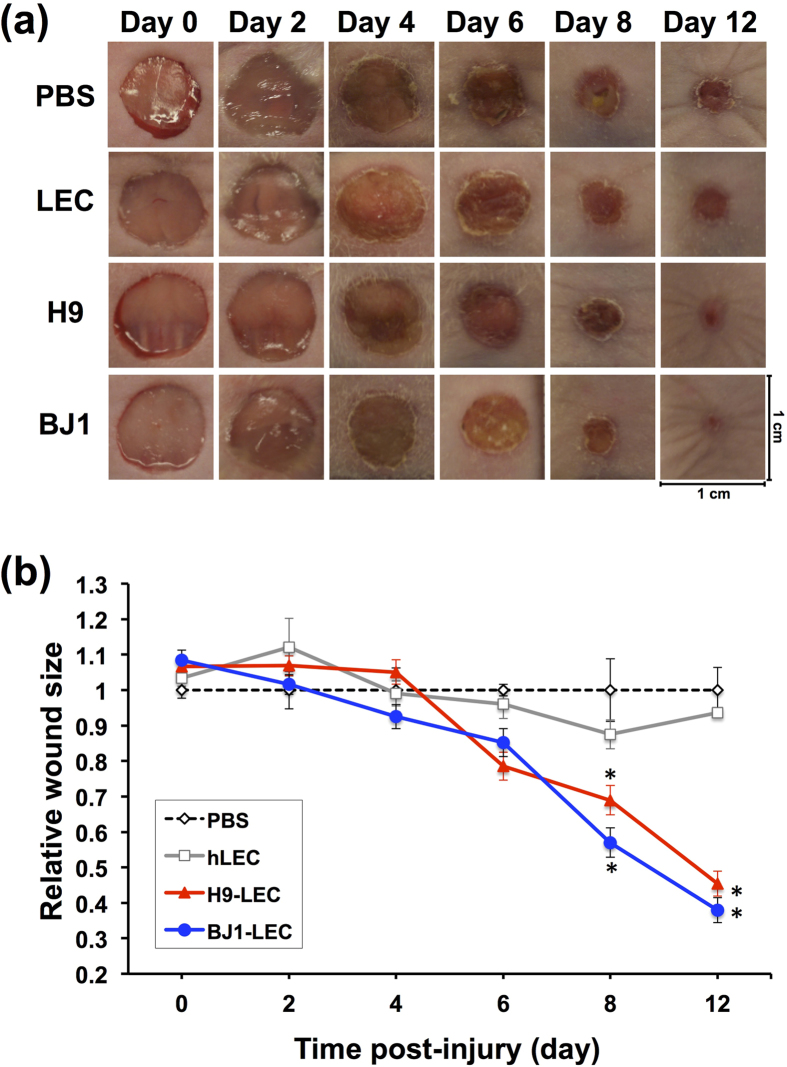
hPSC-derived LECs improve wound healing and increase lymphatic vessel formation. FACS-sorted LYVE-1^+^PODOPLANIN^+^cells derived from hPSCs (H9 and BJ1) were injected into skin wound on the backs of nude mice. PBS and hLECs were used as controls. (**a**) Changes of the wound areas at indicated days after injection. (**b**) Quantitative analysis of wound areas. Y axis represents percent change of wound areas over original wound areas. Note that LYVE-1^+^PODOPLANIN^+^cells derived from both H9 and BJ1 significantly promoted wound closure compared to the PBS- or hLEC-control. Two independent experiments were performed. N = 5 to 9 per group. ^*^P < 0.05, ^**^P < 0.01.

**Figure 6:**
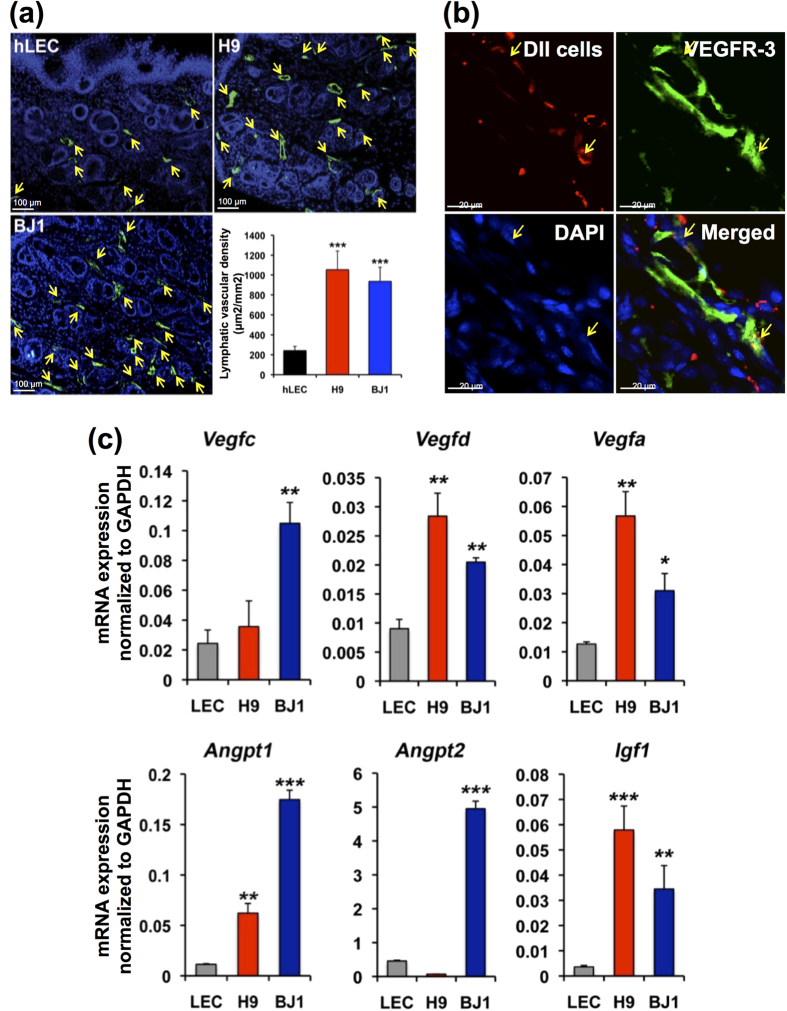
Lymphvasculogenesis and lymphangiogenesis induced by hPSC-LECs in wound healing. (**a**) LYVE-1^+^PODOPLANIN^+^cells derived from hPSCs (H9 and BJ1) were injected into skin wound on the backs of nude mice. Lymphatic vascular density determined at day 14 after cell injection. Lymphatic vessels indicated by arrows (LYVE-1^+^) were significantly increased in mice injected with hPSC-derived LYVE-1^+^PODOPLANIN^+^cells compared to the hLEC-control. ^***^P < 0.001. Polyclonal rabbit anti-LYVE-1 antibody was used for this staining. (**b**) LYVE-1^+^PODOPLANIN^+^cells labeled with CM-Dil were injected into the wounded skin in mice. Confocal microscopic imaging after staining tissues harvested at 2 weeks for VEGFR3 (green) showed incorporation of the injected cells (red) into lymphatic vessels. Arrows indicate co-localization of VEGFR3^+^lymphatic vessels with the injected cells. ^**^P < 0.01. (**c**) Skin tissues from mice injected with hLEC, H9-LEC, or BJ1-LEC were subjected to qRT-PCR analyses. Graphs from 3 independent experiments are shown, ^*^P < 0.05, ^**^P < 0.01, ^***^P < 0.001, N = 3 per group.

Mounting evidence has suggested a crucial role for lymphatic vessel in wound repair^15^,^16^,^17^,^18^,^31^,^32^. Thus, we sought to determine whether the hPSC-derived LECs under our protocol can increase lymphatic neovascularization and enhance wound healing. To this end, we employed a skin wound model which is well known for its involvement with lymphatic vessel growth, and measured the wound area and lymphatic vascular density. LYVE-1^+^PODOPLANIN^+^cells were isolated by FACS at day 14 from differentiating H9 and BJ1 cells under the OP9 co-culture system and injected at four sites (2.5 × 10^4^ cells/site) into the margins of the wound on the backs of nude mice. During the two-week follow-up, mice that had received the LYVE-1^+^PODOPLANIN^+^cells showed enhanced wound healing compared to the PBS or hLEC-injected mice, as demonstrated by decreased area of the wound (Fig. 5a,b). To elucidate the underlying cellular mechanisms for the LEC-mediated wound healing, we conducted immunohistochemical and gene expression analyses with the excised skin 2 weeks after the cell injection. As shown in Fig. 6a, the mice injected with the LYVE-1^+^PODOPLANIN^+^cells exhibited increased lymphatic vessel density compared to the control. Further, the injected cells were clearly incorporated into lymphatic vessels (Fig. 6b), suggesting lymphvasculogenesis. In addition, mice which received hPSC-derived LECs showed a significantly higher expression level of lymphangiogenic factors such as *Vegfc, Vegfd, Vegfa, Angpt1*, and *Angpt2* (Fig. 6c). Together, these results indicate that hPSC-derived LECs can improve wound healing by promoting lymphatic neovascularization through both lymphvasculogenesis and lymphangiogenesis.

## Discussion

This study documents for the first time that pure lymphatic endothelial lineage cells can be generated from differentiating hPSCs, and that these hPSC-derived LECs can effectively repair skin wound by inducing lymphangiogenesis and lymphvasculogenesis *in vivo*.

Several studies have reported lymphatic lineage differentiation from mouse^13^,^28^ or human PSCs^33^. However, no studies have successfully isolated functionally committed (or functional) lymphatic endothelial cells from mouse or human PSCs. As individual LEC markers are redundant in other cell types, confirming co-expression of multiple markers in a given cell is crucial to verify the identity of the PSC-derived LECs. Our study is the first to examine the comprehensive kinetics of multiple LEC marker expression (i.e. *SOX18, COUP-TFII, PROX1, LYVE1, VEGFR3, PODOPLANIN*) during hPSC differentiation, which is critical for studying the human LEC differentiation process as well as for expansion or selection of LECs or LEC precursors. We found that in EB-mediated spontaneous differentiation of hPSCs, LEC markers appeared by day 7 in culture, peaked around 20 days, and were expressed for at least 30 days, whereas previous studies using mESCs showed appearance of LEC markers only after more than 10 days in culture^12^,^13^. This earlier appearance of LEC markers in human cells may be due to the later developmental stage of hPSCs compared to mESCs^34^. Among three conditions that we tested, the co-culture system was more efficient for lymphatic endothelial differentiation than spontaneous differentiation or a feeder-free system, and importantly, the lymphatic endothelial phenotype was effectively maintained with increasing numbers of target cells for 30 days, which is a definite advantage for various applications. Immunocytochemistry and FACS analysis confirmed co-expression of PROX1, VEGFR3, LYVE-1, and PODOPLANIN, but not CD14, a marker for the monocyte-macrophage lineage, indicating a LEC identity of the hPSC-derived cells in our culture system.

One of the important hurdles of hPSC-derived cells for therapy is their low yield. As there are no statistical data regarding the efficiency of lymphatic differentiation of hPSCs, we are unable to compare our system to other lymphatic endothelial differentiation systems. However, compared to the current published data regarding blood vascular endothelial lineage differentiation from human or mouse PSCs, which shows at most 10% of a single marker expression before cell sorting^35^,^36^,^37^,^38^,^39^,^40^,^41^,^42^, the efficiency of our system for lymphatic endothelial cell lineage differentiation was much higher, demonstrated by a single marker expression at more than 40% except for VEGFR3 (10–15%) and expression of the four LEC markers at ~10%. This high yield of differentiation makes this system appealing for testing *in vivo* behavior and therapeutic potential of hPSC-derived LECs.

The LYVE-1^+^PODOPLANIN^+^cells isolated from these lymphatic-differentiated hPSCs demonstrated genuine characteristics of lymphatic endothelial cells as well as progenitor cell features. While fully mature hLECs do not possess therapeutic potential due to their low lymphvasculogenic and lymphangiogenic activities, these purified hPSC-LECs showed such progenitor cell activities and were therapeutically effective. The purified hPSC-LECs expressed all representative LEC markers and were capable of forming tubes, which are typical characteristics of mature LECs. In addition, these hPSC-LECs possessed the capability of being incorporated into lymphatic vessels after transplanted *in vivo* suggesting lymphvasculogenic capability and have high pro-lymphangiogenic capacity. Moreover, when implanted into the skin wound, they were able to induce cutaneous lymphatic neovascularization and promote wound healing with concomitant increase in lymphangiogenic growth factors, indicating that the injected LECs could enhance new lymphatic vessel formation through lymphvasculogenesis and lymphangiogenesis. These findings strongly suggest therapeutic potential of the hPSC-derived LECs in treating diseases related to lymphatic dysfunction. In this regard, growing evidence has suggested that lymphatic vessels can play an important role in wound healing. Saaristo *et al*.^17^ have shown that VEGFC promotes wound healing with concomitant enhancement of lymphangiogenesis as well as angiogenesis in diabetic db/db mice. Maruyama *et al*.^18^ have reported that decreased numbers and activation of macrophages are responsible for reduced lymphatic vessel formation and contribute to impaired diabetic wound healing. It was also shown that COMP-angiopoietin 1 significantly improved skin wound closure, accompanied by increased lymphatic vessel formation in diabetic db/db mice^31^. Inhibition of Notch signaling with Dll1 neutralizing antibodies was shown to impair lymphatic regeneration as well as wound closure^32^. While our study further confirmed that increased lymphatic vessels can promote wound healing, this is the first study to demonstrate that lymphatic vessel growth can be induced by provision of stem cell-derived LECs, highlighting the potential therapeutic utility of the cells generated by our newly developed protocol. Recent studies have suggested that enhanced lymphangiogenesis upon inflammation functions to resolve the tissue edema and facilitate recruitment of immune cells such as macrophages and dendritic cells^43^,^44^,^45^. Wound healing is a representative inflammatory model and optimal wound healing requires a balance between inflammatory cell infiltration and exit. Our data suggest that lymphatic vessel growth induced by hPSC-LEC implantation in the skin can increase drainage of tissue fluid and maintain the balance of inflammatory cells, thereby enhancing wound repair. While this study provides the proof of concept regarding the utility of hPSC-derived LECs in promoting wound healing, it should be validated further in wound healing associated to risk factors favoring wound.

In summary, we developed a novel culture-isolation system to efficiently generate lymphatic endothelial lineage cells from hPSCs. Through this system, cells expressing multiple LEC markers such as PROX-1, LYVE-1, VEGFR3 and PODOPLANIN, but not CD14, were generated at high yield, strongly arguing the LEC identity of the cells. Upon injection into a mouse model of wound, the FACS-sorted LYVE-1^+^PODOPLANIN^+^cells from lymphatic-differentiated hPSCs were able to contribute to the newly developing lymphatic vessels and also promoted lymphatic neovascularization and wound repair. Together, this study for the first time reports the generation of functional LECs from hESCs and hiPSCs. This system could be used as an important platform for investigating human lymphatic vascular differentiation or development and allow generation of lymphatic endothelial cells to be used for cell therapy for intractable wounds or lymphedema.

## Methods

All animal protocols were reviewed and approved by the Emory University Institutional Animal Care and Use Committee and all animal procedures were performed in accordance with the approved guidelines and regulations.

### Cell culture and hESC/iPSC differentiation

H1 and H9 (hESCs) and BJ1 (hiPSCs obtained from Dr. Daley)^19^ were maintained on mitomycin C-treated STO cells in ESC medium as described previously^46^,^47^. To induce spontaneous differentiation via EB formation, mechanically dissociated clumps of hESCs or hiPSCs were cultured in hESC medium without bFGF for the indicated days. For directed differentiation, EBs harvested at day 7 were replated on OP9 cells in 5% FBS/α-MEM supplemented with VEGF-A (50 ng/ml), VEGF-C (100 ng/ml) and EGF (10 ng/ml).

### Fluorescence activated cell sorting (FACS) and Magnetic activated cell sorting (MACS)

EBs differentiated for 7 days under suspension conditions were further cultured on OP9 cells for an additional 14 days. The resulting cells were stained with APC-conjugated sheep anti-human PODOPLANIN (R&D Systems) and biotinylated rabbit anti-human LYVE-1 (ReliaTech GmbH) followed by streptavidin-Alexa 488 (Invitrogen), and then sorted with FACSVantageSE (BD). The purity of the sorting was approximately 98%. For sorting of LYVE-1^+^PODOPLANIN^+^with magnetic-labeled cell separation system (MACS^®^), differentiated hPSCs were incubated with APC-conjugated sheep anti-human PODOPLANIN (R&D Systems) and biotinylated rabbit anti-human LYVE-1 (ReliaTech GmbH). After washing, the cell pellet was incubated with anti-APC and anti-Biotin Microbeads (Miltenyi Biotec) and subjected to MACS sorting (Miltenyi Biotec).

### Wound healing model

hPSC-derived LYVE-1^+^PODOPLANIN^+^cells were isolated using magnetic columns (MACS®, Milteny Biotec). Full-thickness excisional skin wounds (8 mm) were created on the backs of male athymic nude mice, and PBS (100 μl), LYVE-1^+^PODOPLANIN^+^cells (1 × 10^5^) or hLECs (HMVEC-dLy-neo, Lonza) labeled with DiI were injected into the wound bed around the wound. The wound tissues, including a perimeter of 1 to 2 mm of normal skin tissue, were harvested for immunohistochemistry at day 4, 7, or 14 after the injection. Wound closure was monitored at days 0, 2, 4, 6, 8 and 12 and calculation of the area of the wound was performed with the NIH Image J analyzer.

### Ear wound model

After shaving hairs and aseptic preparation, full-thickness excisional skin wounds were created on ears of the mice using 2-mm skin biopsy punches (Baker Cummins Dermatological, Livingston, NJ). hPSC-derived LYVE-1^+^PODOPLANIN^+^cells (1 × 10^5^) labeled with DiI in 50 μl PBS were then injected into and around the wound. Two weeks later, ear tissues were harvested, fixed with 4% PFA, and sectioned for immunohistochemistry.

### Matrigel^®^ tube formation assay

Basement membrane matrix (Matrigel^®^, BD Biosciences) was added to 2-well chamber slides and solidified by incubation at 37 °C for 30 minutes. hPSC-derived LYVE-1^+^PODOPLANIN^+^cells (2 × 10^4^) were labeled with CM-DiI, plated with hLECs (HMVEC-dLy-neo, 1 × 10^5^, Lonza) onto Matrigel, and cultured under α-MEM medium containing 10% FBS, 1% nonessential amino acids, 0.1 mM β-mercaptoethanol, 4 ng/mL of FGF2, 10 ng/ml VEGF-A, 20 ng/mL of VEGF-C, and 10 ng/ml EGF, and incubated at 37 °C for 12 h. After removing the medium, 4% PFA was added for fixation. The tube structures were evaluated by microscopy.

### Immunocytochemistry and Immunohistochemistry

Cells were fixed with 4% PFA for 30 min at 4 °C. After washing with PBS, the cells were blocked with 5% serum and subjected to staining with primary antibodies followed by secondary antibodies. DAPI was used for nuclear staining and the cells were visualized under a fluorescent microscope (Nikon). Primary antibodies: mouse anti-human PODOPLANIN D2-40 (Covance), rabbit anti-human PROX-1 (Abcam), rabbit biotin conjugated anti-human PROX-1 (ReliaTech GmbH), rabbit anti-LYVE-1 (ReliaTech GmbH), and rabbit anti-VEGFR3 (Santa Cruz Biotechnology). The LYVE-1 and VEGFR3 antibodies have cross-reactivity to human, mouse, and rat. Secondary antibodies: FITC-conjugated goat anti-rabbit IgG, Cy3-conjugated goat anti-mouse IgG (Jackson Immunoresearch Laboratories), Alexa Fluor® 647 conjugated anti-mouse IgG, or Alexa Fluor® 488 conjugated Streptavidin (Molecular Probes). For immunohistochemistry, samples frozen in OCT embedding medium (Sakura) were sectioned. The 6 to 30 μm tissue sections were blocked with 3% normal goat serum in 0.1% Triton-X100 in phosphate-buffered saline (PBS) for 2 hours and were incubated overnight with primary antibodies followed by secondary antibodies at 4 °C. Images were taken with a LSM 510 Meta confocal laser scanning microscope (Carl Zeiss).

### Flow cytometry

FACS staining and analysis were performed as previously described^29^. For four-marker staining, cells were incubated with Alexa488-conjugated rat anti-human PODOPLANIN (Biolegend), PE-conjugated mouse anti-human VEGFR3 (R&D system), and biotinylated rabbit anti-human LYVE-1 (ReliaTech GmbH) followed by streptavidin PE-Cy5 (BD Pharminogen). After washing, the cells were fixed/permeabilized (IC Fixation/Permeabilization buffer, eBioscience) and incubated with goat anti-human PROX-1 (R&D Systems), followed by donkey APC-Cy7-conjugated anti-goat IgG (Santa cruz). The resulting cells were analyzed on a C6 Flow Cytometer (Accuri Cytometers Inc) and data were evaluated with CFlow software. For single marker staining, we used the same antibodies as described.

### Quantitative RT-PCR

Total RNA extraction and cDNA synthesis was performed as previously described^48^. Briefly, total RNA was extracted from cells or tissues using the RNeasy kit (Qiagen) according to the manufacturer’s instructions. Subsequently, extracted RNA was reverse-transcribed using Taqman Reverse Transcription Reagents (Applied Biosystems) according to the manufacturer’s instructions. The synthesized cDNA was subjected to qPCR using specific primers and probes. Quantitative assessment of RNA levels was performed using an ABI PRISM 7500 Sequence Detection System (Applied Biosystems). Relative mRNA expression normalized to *GAPDH* expression was calculated by using the formula Relative Expression Level = 2^−ΔCT^, where ΔCT = CT gene of interest – CT GAPDH. The primers and probes were designed using Primer Express 3.0 (Applied Biosystems) and listed in Supplementary information, Table S1.

### Statistical analyses

All results were expressed as mean ± SEM. Statistical analysis was performed by an unpaired Student’s t-test for comparisons between two groups and two-way repeated measures ANOVA, and Bonferroni-post comparison test was used for the results in Fig. 5b comparing three groups. P value < 0.05 was considered to denote statistical significance.

## Additional Information

**How to cite this article**: Lee, S.-J. *et al*. Generation of pure lymphatic endothelial cells from human pluripotent stem cells and their therapeutic effects on wound repair. *Sci. Rep*. **5**, 11019; doi: 10.1038/srep11019 (2015).

## Supplementary Material

Supplementary Information
