# Supplementary material for: Generation of pure lymphatic endothelial cells from human pluripotent stem cells and their therapeutic effects on wound repair
Source: Sci Rep. 2015 Jun 12;5:11019. doi: 10.1038/srep11019 (PMC4464258; doi:10.1038/srep11019)
Supplement: Supplementary Information [file srep11019-s1.doc]

#### Supplementary Information

**Generation of pure lymphatic endothelial cells from human pluripotent stem cells and their therapeutic effects on wound repair**

Shin-Jeong Lee, Changwon Park, Ji Yoon Lee, Sangsung Kim, Pil Jae Kwon, Woansang Kim, Yong Heui Jeon, Eugine Lee, and Young-sup Yoon


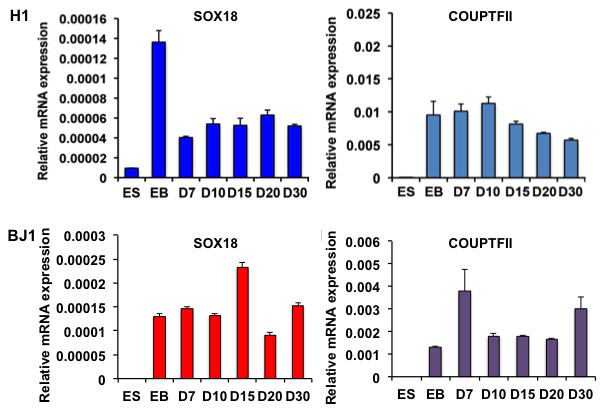


**Supplementary Figure** **S1.** Expression of early lymphatic markers, SOX18 and COUP-TFII in the differentiating hPSC (H1 and BJ1). EBs differentiated for 7 days in suspension culture were replated on OP9 cells, further cultured for an additional 30 days with VEGF-A, -C and EGF, and subjected to qRT-PCR.


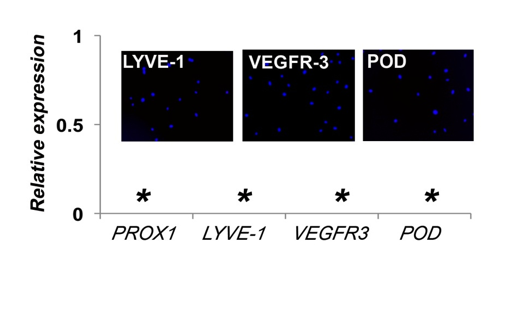


**Supplementary Figure** **S2.** OP9 cells do not express LEC markers such as PROX-1, LYVE-1, VEGFR-3 and PODOPLANIN. OP9 cells were subjected to immunocytochemistry and qRT-PCR analysis for LEC markers. *ND: non-detected. Inserts display the immunocytochemistry results of DAPI-positive OP9 cells.

**
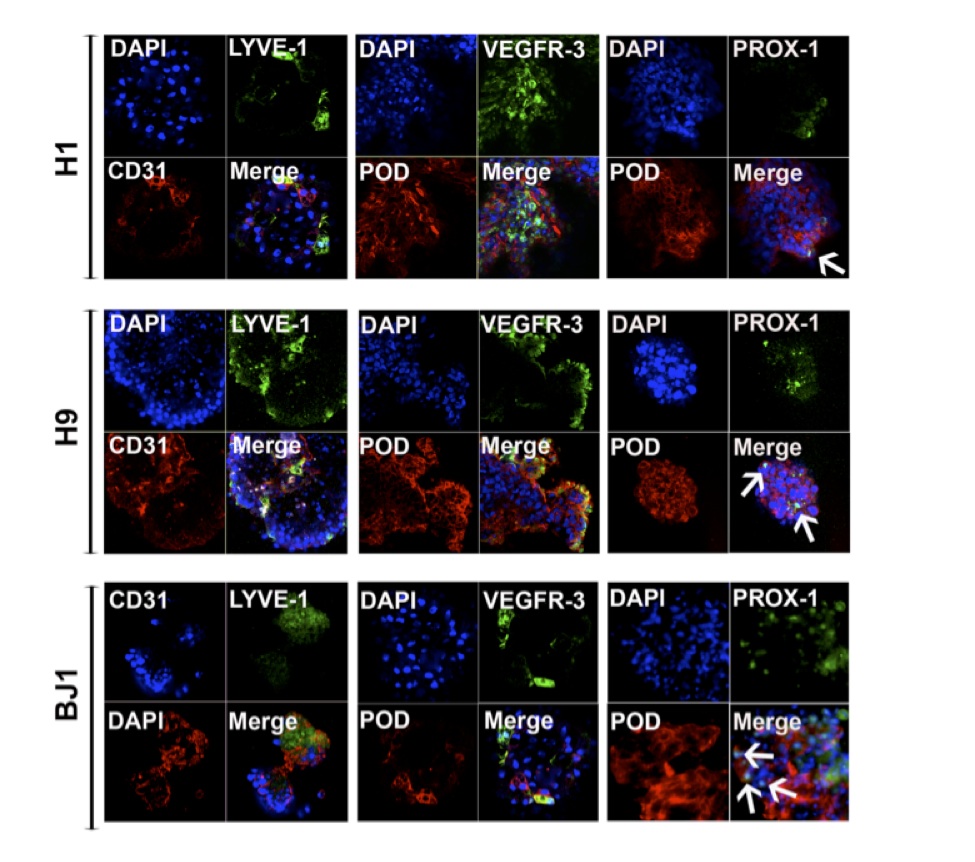
**

**Supplementary Figure** **S3.** Immunocytochemistry for expression of LEC markers in differentiating hESC (H1 and H9) and hiPSC (BJ1) lines. EBs harvested between 10 and 15 days of spontaneous differentiation were subjected to immunocytochemistry with antibodies against LEC markers. Arrows indicate cells stained positive for PROX-1.


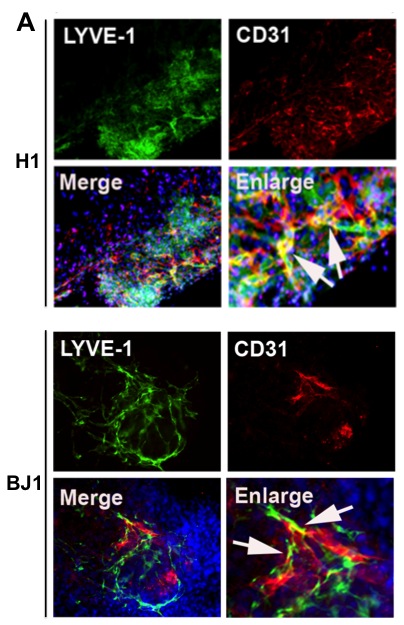


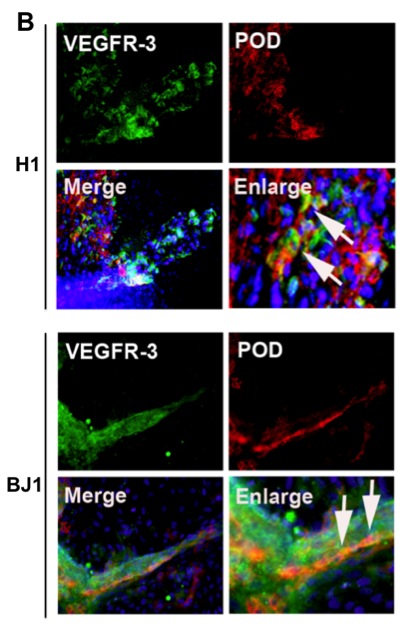


**
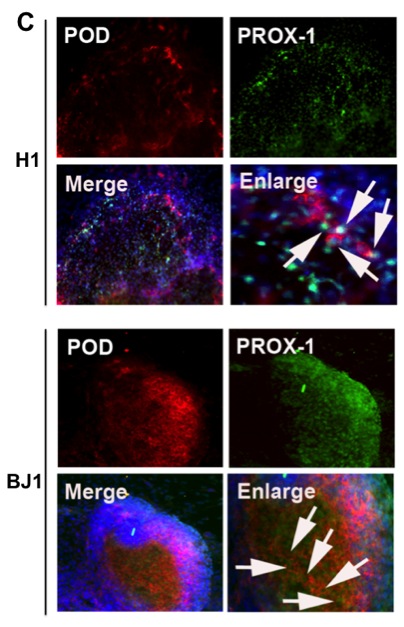
**

**Supplementary Figure** **S4.** Immunocytochemistry showing expression of LEC markers in the differentiated hESCs and hiPSCs. The pluripotent stem cells differentiated in suspension culture were subsequently cultured for 10 to 15 days under the co-culture conditions. Double LEC marker staining for LYVE-1 and CD31, VEGFR-3 and PODOPLANIN (B), and PODOPLANIN and PROX-1 (C). Arrows indicate the cells stained positive for two LEC markers as labeled.


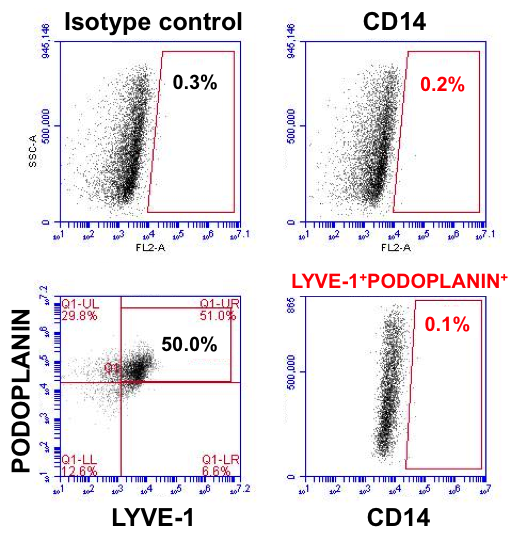


**Supplementary Figure** **S5.**  hESC-derived LYVE-1^+^PODOPLANIN^+^ cells do not express a monocyte-macrophage marker, CD14. The hESCs (H1) differentiated for 14 days were subjected to FACS analysis for PODOPLANIN, LYVE-1, and CD14. Number in each box represents percentage of positive cells of each indicated protein.


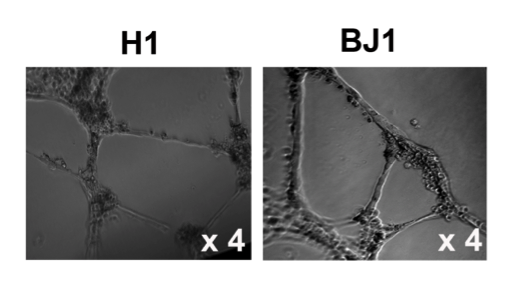


**Supplementary Figure** **S6.** The sorted LYVE-1^+^ PODOPLANIN^+^ cells form tube-like structures. Cells from the OP9 co-culture were sorted and subjected to a Matrigel tube formation assay. Images were taken 10 days after the replating.

**Supplementary Table S1**. Primers and probes for quantitative RT-PCR

| **Human-specific** | **Forward** | **Reverse** | **Probe** |
| --- | --- | --- | --- |
| PRROX1 | GCCAGATTTGCAGTCAATGG | ATGATGACGTCGCCAAAGC | TTTCCACACCGCCAAC |
| LYVE-1 | CTGGGTTGGAGATGGATTCG | TCAGGACACCCACCCCATTT | TAGCCCAAACCCCAAGTG |
| VEGFR3 | CCTTGCCCGGGACATCTA | TTGTCGAAGATGCTTTCAGGG | AGACCCCGACTACGTCCGCAAGG |
| PODOPLANIN | CAGGTGCCGAAGATGATGTG | TGTTGCCACCAGAGTTGTCA | TGACTCCAGGAACCAG |
| PECAM1 | TGTATTTCAAGACCTCTGTGCACTT | TTAGCCTGAGGAATTGCTGTGTT | TTTATGAACCTGCCCTGCTCCCACA |
| FGF2 | AGCGACCCTCACATCAAGCTA | CCAGGTAACGGTTAGCACACACT | TTGATAGACACAACTCCTCTCTCTTCTGCTTGAAGT |
| HGF | CAATAGTCAATTTAGACCATCCCGTAAT | CGTGTTGGAATCCCATTTACAA | CTTGTGCCAAAACGAAACAATTGCGA |
| IGF1 | CCATGTCCTCCTCGCATCTC | CGTGGCAGAGCTGGTGAAG | ACCTGGCGCTGTGCCTGCTCA |
| ANGPT1 | CAGAAAACAGTGGGAGAAGATATAACC | TGCCATCGTGTTCTGGAAGA | TCAACATGGGCAATGTGCCTACACTTTC |
| GAPDH | GGTGGTCTCCTCTGACTTCAACA | GTGGTCGTTGAGGGCAATG | GTGGTCGTTGAGGGCAATG |
| **Mouse-specific** | **Forward** | **Reverse** | **Probe** |
| Vegfa | CATCTTCAAGCCGTCCTGTGT | CAGGGCTTCATCGTTACAGCA | CCGCTGATGCGCTGTGCAGG |
| Fgf2 | GTCACGGAAATACTCCAGTTGGT | CCGTTTTGGATCCGAGTTTATACT | TGTGGCACTGAAACGAACTGGG |
| Igf1 | TGCTTCCGGAGCTGTGATCT | CGGGCTGCTTTTGTAGGCT | AGGAGACTGGAGATGTACTGTGCCCCAC |
| Angpt1 | GGGACAGCAGGCAAACAGA | TGTCGTTATCAGCATCCTTCGT | TTGATCTTACACGGTGCCGATT |
| Angpt2 | TCAGCCAACCAGGAAGTGATT | AGCATCTGGGAACACTTGCAG | CACAAAGGATTCGGACAATGACAAATGCA |
| Vegfc | CAGCAAGACGTTGTTTGAAATTACA | GTGATTGGCAAAACTGATTGTGA | CCTCTCTCACAAGGCCCCAAACCA |
| Gapdh | CGTGTTCCTACCCCCAATGT | TGTCATCATACTTGGCAGGTTTCT | TCGTGGATCTGACGTGCCGCC |
